# Supplementary material for: Skin Immuno-CometChip in 3D vs. 2D Cultures to Screen Topical Toxins and Skin-Specific Cytochrome Inducers
Source: Genes (Basel). 2023 Mar 2;14(3):630. doi: 10.3390/genes14030630 (PMC10048716; doi:10.3390/genes14030630)
Supplement: Supplementary file 1 [file genes-14-00630-s001.zip › genes-2235298-supplementary.docx]

Supplemental Material:

***Figure S1.*** ***Validation of DNA damage and freeze protocol to develop DNA damage control cells.***

***Figure S1 Validation of DNA damage and freeze protocol* (A)** Dose-response curve for etoposide in HaCaT cells after 60 min exposure. Assay lost linearity with higher concentrations of etoposide**.** **(B)** Only concentrations between 0- 10 µM etoposide were used in DNA damage control experiments **(C)** HaCaT cells maintained approximately 40% DNA damage after 60 min exposure to 10 μM etoposide. Cells were stored at -80°C for the times indicated on X-axis. **(D)** DNA damage levels from 10 separate CometChip assays performed after three months of storage. Error bars represent ±SEM, data points represent technical replicates from frozen stocks. n= 254-618 individual comets assays (in A, B).

***Figure S2. PBS washes eliminate β1-integrin negative Jurkat cells***

**(C)**

***Figure S2. Two PBS washes eliminate β1-integrin negative Jurkat cells (A, left), but not β1-integrin positive basal keratinocytes (A, middle) from CometChip microwells*,** visualized by biotinylated anti-β1 integrin and Streptavidin-coupled Qdot 655, or by SYBR Gold staining for DNA (A, right). (B) H_2_O_2_ treatment of EpiDerm organotypic cultures, followed by CometChip electrophoresis reveals that anti-β1 integrin and Streptavidin-coupled Qdot 655 staining does not interfere with comet assay, where DNA tails can be seen as a green streak upon SYBR Gold staining, and DNA damage can be quantified and seen to be dose dependent (C) in basal keratinocytes. Magnification: 4X, Fluorescence: green & red overlay, exposure: 5 s. Results shown are the means ± SD of three replicates of a representative experiment; essentially the same results were obtained in three independent experiments. *, **, *** represent p < 0.05, p < 0.01, and p < 0.001, respectively.

| Table S1: Summary of Genotoxicity, Cytotoxicity, and CYP Induction | |  |  | | | | |  | | |  | |
| --- | --- | --- | --- | --- | --- | --- | --- | --- | --- | --- | --- | --- |
|  |  |  | HaCaT | | | NHEK | 3D | NHEK | HaCaT | HepG2 |  |  |
|  |  | CAS No. | -FBS | +FBS | +S9 |  | Epi |  |  |  | 3D | HaCaT |
| A01 | Benzidine | 92-87-5 |  |  |  |  |  |  |  |  |  |  |
| A02 | 4-4' Methylenedianiline 2B | 101-77-9 |  |  |  |  |  |  |  |  |  |  |
| A03 | Coumarin | 91-64-5 |  |  |  |  |  |  |  |  |  |  |
| A04 | 2 ,3-dichloronitrobenzene | 3209-22-1 |  |  |  |  |  |  |  |  |  |  |
| A05 | 2, 4-dichloronitrobenzene | 611-06-3 |  |  |  |  |  |  |  |  |  |  |
| A06 | 2-hydroxy-4-methoxybenzophenone | 131-57-7 |  |  |  |  |  |  |  |  |  |  |
| A07 | 2 2-bis(bromomethyl)-1 3-propanediol | 3296-90-0 |  |  |  |  |  |  |  |  |  |  |
| A08 | michler's ketone | 90-94-8 |  |  |  |  |  |  |  |  |  |  |
| A09 | 8-methylquinoline | 611-32-5 |  |  |  |  |  |  |  |  |  |  |
| A10 | quinoline | 91-22-5 |  |  |  |  |  |  |  |  |  |  |
| A11 | 7-methyl quinoline | 612-60-2 |  |  |  |  |  |  |  |  |  |  |
| A12 | 6-methyl quinoline | 91-62-3 |  |  |  |  |  |  |  |  |  |  |
| B01 | Zn dibutylDITC diHCl | 3101-60-8 |  |  |  |  |  |  |  |  |  |  |
| B02 | 1-Amino-2-methylanthraquinone | 82-28-0 |  |  |  |  |  |  |  |  |  |  |
| B03 | Benzo[a]pyrene | 50-32-8 |  |  |  |  |  |  |  |  | 1A1 | 1A1 |
| B04 | N-Nitrosodimethylamine | 62-75-9 |  |  |  |  |  |  |  |  |  |  |
| B05 | Cyclophosphamide | 6055-19-2 |  |  |  |  |  |  |  |  |  |  |
| B06 | 2-Naphthylamine | 91-59-8 |  |  |  |  |  |  |  |  |  |  |
| B07 | 3-Acetyl-2,5-dimethylfuran | 10599-70-9 |  |  |  |  |  |  |  |  |  |  |
| B08 | Di(2-methoxyethyl) phthalate | 117-82-8 |  |  |  |  |  |  |  |  |  |  |
| B09 | tert-Butylphenylglycidyl ether | 3101-60-8 |  |  |  |  |  |  |  |  |  |  |
| B10 | Primidone | 125-33-7 |  |  |  |  |  |  |  |  |  |  |
| B11 | Pentachloroanisole | 1825-21-4 |  |  |  |  |  |  |  |  |  |  |
| B12 | 7,12-DMBA | 57-97-6 |  |  |  |  |  |  |  |  |  |  |
| C01 | 2-Acetylaminofluorene | 53-96-3 |  |  |  |  |  |  |  |  |  |  |
| C02 | Cisplatin | 15663-27-1 |  |  |  |  |  |  |  |  |  |  |
| C03 | ENU | 759-73-9 |  |  |  |  |  |  |  |  |  |  |
| C04 | MMS | 66-27-3 |  |  |  |  |  |  |  |  |  |  |
| C05 | EMS | 62-50-0 |  |  |  |  |  |  |  |  |  |  |
| C06 | CdCl2 | 10108-64-2 |  |  |  |  |  | . |  |  | 1B1 |  |
| C07 | Hydroquinone | 123-31-9 |  |  |  |  |  |  |  |  | 1B1 |  |
| C08 | Sodium dichromate(VI) dihydrate | 7789-12-0 |  |  |  |  |  |  |  |  | 1B1 |  |
| C09 | Adriamycin HCl | 25316-40-9 |  |  |  |  |  |  |  |  | 1B1 | ***lower 1A1 |
| C10 | Diglycidyl resorcinol ether | 101-90-6 |  |  |  |  |  |  |  |  | 1B1 |  |
| C11 | Glycidol | 556-52-5 |  |  |  |  |  |  |  |  |  |  |
| C12 | Ampicillin trihydrate | 7177-48-2 |  |  |  |  |  |  |  |  |  |  |
| D01 | D-Mannitol | 69-65-8 |  |  |  |  |  |  |  |  |  |  |
| D02 | Phenformin HCl | 834-28-6 |  |  |  |  |  |  |  |  |  |  |
| D03 | n-Butyl Chloride | 109-69-3 |  |  |  |  |  |  |  |  |  |  |
| D04 | (2-Chloroethyl)trimethyl-ammonium chloride | 999-81-5 |  |  |  |  |  |  |  |  |  |  |
| D05 | Cyclohexone | 108-94-1 |  |  |  |  |  |  |  |  |  |  |
| D06 | N.N'-dicyclohexylthiourea | 1212-29-9 |  |  |  |  |  |  |  |  |  |  |
| D07 | Fluometron | 2164-17-2 |  |  |  |  |  |  |  |  |  |  |
| D08 | D-Limonene | 5989-27-5 |  |  |  |  |  |  |  |  |  |  |
| D09 | Di-(2-ethylhexyl)phthalate | 117-81-7 |  |  |  |  |  |  |  |  |  |  |
| D10 | Melamine | 108-78-1 |  |  |  |  |  |  |  |  |  |  |
| D11 | Progesterone | 57-83-0 |  |  |  |  |  |  |  |  | 1A1 |  |
| D12 | Tris(2-ethylhexyl)phosphate | 78-42-2 |  |  |  |  |  |  |  |  |  |  |
| E01 | Urea | 57-13-6 |  |  |  |  |  |  |  |  |  |  |
| E02 | Curcumin | 458-37-7 |  |  |  |  |  |  |  |  | 1A1 |  |
| E03 | Phthalic anhydride | 85-44-9 |  |  |  |  |  |  |  |  |  |  |
| E04 | Benzyl alcohol | 100-51-6 |  |  |  |  |  |  |  |  |  |  |
| E05 | 2Chloroethyldiethylammonium chloride | 869-24-9 |  |  |  |  |  |  |  |  | 1A1 | 1A1 |
| E06 | Zinc dibutyldithiocarbamate | 136-23-2 |  |  |  |  |  | . |  |  |  | 1A1 |
| E07 | 17beta-Estradiol | 50-28-2 |  |  |  |  |  |  |  |  |  |  |
| E08 | Bisphenol A | 80-05-7 |  |  |  |  |  |  |  |  |  |  |
| E09 | Dipyrithione | 3696-28-4 |  |  |  |  |  |  |  |  |  |  |
